# Sufficient conditions for oscillation of a nonlinear fractional nabla difference system

**DOI:** 10.1186/s40064-016-2820-2

**Published:** 2016-07-26

**Authors:** Wei Nian Li, Weihong Sheng

**Affiliations:** Department of Mathematics, Binzhou University, Binzhou, 256603 Shandong People’s Republic of China

**Keywords:** Oscillation, Nonlinear fractional nabla difference equation, Discrete fractional calculus, 26A33, 39A12, 39A21

## Abstract

In this paper, we study the oscillation of nonlinear fractional nabla difference equations of the form

$$ \left\{ \begin{array}{ll}\nabla _a^{\alpha }x(t)+q(t)f(x(t))=g(t), &{}\quad t\in {\mathbb {N}}_{a+1},\\ \nabla _a^{-(1-\alpha )}x(t)|_{t=a}=c, \end{array}\right. $$

where *c* and *α* are constants, $0<\alpha <1,\nabla _a^{\alpha }$ is the Riemann–Liouville fractional nabla difference operator of order $\alpha , a\ge 0$ is a real number, and ${\mathbb {N}}_{a+1}=\{a+1,a+2,\ldots \}$. Some sufficient conditions for oscillation are established.

## Background

It is well known that discrete fractional calculus is a very new area. In 1988, Miller and Ross (1989) firstly introduced the definitions of non-integer order differences and sums. Since then, several authors started to study the theory of fractional difference equations. Especially, some excellent results have been established in recent years. For example, we refer the reader to Atici and Eloe (2007, 2009a, 2009b, 2011, 2012), Abdeljawad and Atici (2012), Atici and Wu (2014), Goodrich (2011, 2012), Anastassiou (2010, 2011), Čermák et al. (2015), Dassios and Baleanu (2013, 2015), Dassios et al. (2014), Hein et al. (2011), Abdeljawad (2011, 2013a, b), Alzabut and Abdeljawad (2014), Kang et al. (2014), Diblík (2015), Kisalar et al. (2015), Erbe et al. (2016), Li (2016) and the references therein.

The oscillation theory is an important part of the qualitative theory of fractional difference equations. However, to the best of our knowledge, few papers (Alzabut and Abdeljawad 2014; Kisalar et al. 2015; Li 2016) are known regarding the oscillatory behavior of fractional difference equations.

In Alzabut and Abdeljawad (2014), Alzabut and Abdeljawad considered the oscillation for nonlinear fractional difference equation of the form

$$ \left\{ \begin{array}{lll} \nabla _{a(q)-1}^{q}x(t)+f_1(t,x(t))=r(t)+f_2(t,x(t)), &{}\quad t\in {\mathbb {N}}_{a(q)}, \\ \nabla _{a(q)-1}^{-(1-q)}x(t)|_{t=a(q)}=x(a(q))=c, &{}\quad c\in {\mathbb {R}}, \end{array}\right. \quad \quad \quad \quad \quad \quad  (E1) $$

where ${\mathbb {N}}_{a(q)}=\{a(q)+1,a(q)+2,\ldots \}, \ a(q)=a+m-1, \ m=[q]+1, m-1<q<m, \ m\in {\mathbb {N}}, r:{\mathbb {N}}_{a(q)}\rightarrow {\mathbb {R}}, f_i:{\mathbb {N}}_{a(q)}\times {\mathbb {R}}\rightarrow {\mathbb {R}}, (i=1,2), \nabla _{a(q)}^q$ is the Riemann–Liouville fractional nabla difference operator of order *q* of *x*, and $\nabla _{a(q)}^{-q}$ is *q*-th fractional sum operator.

In Kisalar et al. (2015), Kisalar et al. studied oscillatory behavior of the higher order fractional nonlinear difference equations of the form

$$ \left\{ \begin{array}{lll} \Delta ^{\alpha }x(t)+f_1(t,x(t+\alpha ))=v(t)+f_2(t,x(t+\alpha )), &{}\quad t\in {\mathbb {N}}_{0}, \ m-1<\alpha \le m, \\ \Delta ^{\alpha -k}x(t)|_{t=0}=x_k, &{}\quad k=1,2,\ldots ,m-1, \end{array}\right. \quad \quad  (E2) $$

where $\Delta ^{\alpha }$ is a Riemann–Liouville fractional delta difference operator of order *α*, $m-1<\alpha \le m,m\ge 1$ is an integer, $f_i: [0,\infty )\times {\mathbb {R}} \rightarrow {\mathbb {R}}, (i=1,2)$ and *v* are continuous with respect to *t* and $x,{\mathbb {N}}_a = \{a, a+1, a+2, \ldots \}$.

In Li (2016), Li established the oscillation of forced fractional difference equations with damping term of the form

$$ (1+p(t))\Delta (\Delta ^{\alpha }x(t))+p(t)\Delta ^{\alpha }x(t)+f(t,x(t))=g(t), \quad t\in {\mathbb {N}}_0, \qquad \qquad \qquad \qquad (E3) $$

with initial condition $\Delta ^{\alpha -1}x(t)|_{t=0}=x_0,$ where $0<\alpha <1$ is a constant, $\Delta ^{\alpha }x$ is the Riemann–Liouville fractional delta difference operator of order *α* of *x*, and ${\mathbb {N}}_0=\{0,1,2,\ldots \}$.

In Atici and Eloe (2012), Atici and Eloe considered the following initial value problem for a nonlinear fractional difference equation

$$ \left\{ \begin{array}{lll} \nabla _{a}^{\nu }x(t)=f(t,x(t)), &{}\quad \text{ for }\ \ t=a+1,a+2,\ldots , \\ \nabla _{a}^{-(1-\nu )}x(t)|_{t=a}=x(a)=c, \end{array}\right. \qquad \qquad \qquad \qquad \qquad (E4)$$

where $0<\nu \le 1$ and *a* is any real number. The authors obtained that *x*(*t*) is a solution of (*E*4) if and only if

$$ x(t)=\frac{(t-a+1)^{\overline{\nu -1}}}{\Gamma (\nu )}x(a)+\nabla _{a+1}^{-\nu }f(t,x(t)). $$

Motivated by the papers (Atici and Eloe 2012; Alzabut and Abdeljawad 2014; Kisalar et al. 2015; Li 2016), in this paper, we investigate the oscillation of a nonlinear fractional nabla difference system of the form

1 $$ \left\{ \begin{array}{ll} \nabla _a^{\alpha }x(t)+q(t)f(x(t))=g(t), &{}\quad t\in {\mathbb {N}}_{a+1}, \\ \nabla _a^{-(1-\alpha )}x(t)|_{t=a}=c,\end{array}\right. $$

where *c* and *α* are constants, $0<\alpha <1,\nabla _a^{\alpha }$ is the Riemann–Liouville fractional nabla difference operator of order $\alpha ,a\ge 0$ is a real number, and ${\mathbb {N}}_{a+1}=\{a+1,a+2,\ldots \}$.

In this paper, we always assume that

(A) $f:{\mathbb {R}}\rightarrow {\mathbb {R}}$, and $xf(x)>0$ for $x\ne 0,g:{\mathbb {N}}_{a+1}\rightarrow {\mathbb {R}}$, and $q(t)\ge 0,t\in {\mathbb {N}}_{a+1}$.

A solution *x*(*t*) of the system (1) is said to be oscillatory if it is neither eventually positive nor eventually negative, otherwise it is nonoscillatory.

## Preliminaries

In this section, we collect some basic definitions and lemmas that will be important to us in what follows. For an excellent introduction to the discrete fractional calculus, we refer the reader to papers (Atici and Eloe 2009a, 2012; Abdeljawad and Atici 2012; Anastassiou 2010, 2011; Abdeljawad 2011, 2013b).

### **Definition 1**

(Atici and Eloe 2012) Let $\nu >0$. The $\nu$-th fractional sum *f* is defined by

2 $$ \nabla _a^{-\nu }f(t)=\frac{1}{\Gamma (\nu )}\sum _{s=a}^{t}(t-s+1)^{\overline{\nu -1}}f(s), $$

for $t\in {\mathbb {N}}_a=\{a,a+1,a+2,\ldots \}$, where $\Gamma$ is the gamma function, and

3 $$ t^{\overline{\nu }}=\frac{\Gamma (t+\nu )}{\Gamma (t)}. $$

### **Definition 2**

(Atici and Eloe 2012) Let $\mu >0$ and $m-1<\mu <m$, where *m* denotes a positive integer. Set $\nu =m-\mu$. The $\mu$-th fractional nabla difference is defined as

4 $$ \nabla _a^{\mu }f(t)=\nabla _a^{m-\nu }f(t)=\nabla _a^m\nabla _a^{-\nu }f(t).$$

### **Lemma 3**

(Atici and Eloe 2012) *Letfbe a real-valued function defined on*${\mathbb {N}}_a$, *and let*$\mu ,\nu >0$. *Then*

5 $$ \nabla _a^{-\nu }[\nabla _a^{-\mu }f(t)]=\nabla _a^{-(\mu +\nu )}f(t)=\nabla _a^{-\mu }\left[ \nabla _a^{-\nu }f(t)\right] , $$

*and*

6 $$ \nabla _{a+1}^{-\nu }\nabla f(t)=\nabla \nabla _a^{-\nu }f(t)-\frac{(t-a+1)^{\overline{\nu -1}}}{\Gamma (\nu )}f(a). $$

### **Lemma 4**

(Atici and Eloe 2012) *For every*$t\in {\mathbb {N}}_a$,

7 $$ \nabla _a^{-\nu }(t-a+1)^{\overline{\mu }}=\frac{\Gamma (\mu +1)}{\Gamma (\mu +\nu +1)}(t-a+1)^{\overline{\nu +\mu }}. $$

### **Lemma 5**

*Let*

8 $$ E(t)=\sum _{s=a}^{t}(t-s+1)^{\overline{-\alpha }}x(s), \quad t\in {\mathbb {N}}_a. $$

*Then*

9 $$ \nabla E(t)=\Gamma (1-\alpha )\nabla _a^{\alpha }x(t). $$

### Proof

Using Definition 1, it follows from (8) that

10 $$ \begin{aligned} E(t)&=\sum _{s=a}^{t}(t-s+1)^{\overline{-\alpha }}x(s)=\sum _{s=a}^{t}(t-s+1)^{\overline{(1-\alpha )-1}}x(s)\\ &=\Gamma (1-\alpha )\nabla _a^{-(1-\alpha )}x(t).\end{aligned} $$

Using Definition 2, it follows from (10) that

$$ \nabla E(t)=\Gamma (1-\alpha )\nabla \nabla _a^{-(1-\alpha )}x(t)=\Gamma (1-\alpha )\nabla _a^{\alpha }x(t). $$

The proof of Lemma 5 is complete.

### **Lemma 6**

*Let*$a\ge 0$*and*$0<\alpha <1$*be real number*, $u,v:{\mathbb {N}}_a\rightarrow {\mathbb {R}}$. *If*

11 $$ u(t)\le v(t), \quad t\in {\mathbb {N}}_a, $$

then

12 $$ \nabla _a^{-\alpha }u(t)\le \nabla _a^{-\alpha }v(t). $$

### Proof

It follows from (11) that

13 $$ (t-s+1)^{\overline{\alpha -1}}u(s)\le (t-s+1)^{\overline{\alpha -1}}v(s) \quad \text{ for } \ \ s=a,a+1,\ldots ,t. $$

Summing both sides of (13) from *a* to *t*, we have

14 $$ \sum _{s=a}^t(t-s+1)^{\overline{\alpha -1}}u(s)\le \sum _{s=a}^t(t-s+1)^{\overline{\alpha -1}}v(s). $$

Using Definition 1, from (14) we easily obtain (12). This completes the proof of Lemma 6.

## Main results

In this section, we establish the oscillation results of system (1).

### **Theorem 7**

*Assume that*

15 $$ \liminf _{t\rightarrow \infty }\left\{ (t-a)^{1-\alpha }\sum _{s=a+1}^{t}(t-s+1)^{\overline{\alpha -1}}g(s)\right\} =-\infty ,$$

*and*

16 $$ \limsup _{t\rightarrow \infty }\left\{ (t-a)^{1-\alpha }\sum _{s=a+1}^{t}(t-s+1)^{\overline{\alpha -1}}g(s)\right\} =+\infty . $$

*Then every solutionx*(*t*) *of the system* (1) *is oscillatory.*

### Proof

Suppose to the contrary that there is a nonoscillatory solution *x*(*t*) of system (1). It is obvious that there exists $t_0\in {\mathbb {N}}_{a+1}$ such that $x(t)>0$ or $x(t)<0, \ t\ge t_0.$

### *Case 1*

$x(t)>0, \ t\ge t_0.$ Noting the assumption (A), from the system (1) , we have

17 $$ \nabla _a^{\alpha }x(t)=-q(t)f(x(t))+g(t)\le g(t). $$

Using Lemma 6, from (17), we have

18 $$\nabla _{a+1}^{-\alpha }\nabla _a^{\alpha }x(t)\le \nabla _{a+1}^{-\alpha }g(t). $$

Using Definition 2, Lemma 3 in the left-hand side of (18) and noting the initial condition of system (1) , we obtain

19 $$\begin{aligned} \nabla _{a+1}^{-\alpha }\nabla _a^{\alpha }x(t) &=\nabla _{a+1}^{-\alpha }\nabla \nabla _a^{-(1-\alpha )}x(t)\\ &=\nabla \nabla _a^{-\alpha }\nabla _a^{-(1-\alpha )}x(t) -\frac{(t-a+1)^{\overline{\alpha -1}}}{\Gamma (\alpha )} \nabla _a^{-(1-\alpha )}x(a)\\ &=x(t)-\frac{c}{\Gamma (\alpha )}(t-a+1)^{\overline{\alpha -1}}. \end{aligned}$$

Using Definition 1, it follows from the right-hand side of (18) that

20 $$ \nabla _{a+1}^{-\alpha }g(t) =\frac{1}{\Gamma (\alpha )}\sum _{s=a+1}^{t}(t-s+1)^{\overline{\alpha -1}}g(s). $$

Combining (18)–(20), we have

21 $$ x(t)\le \frac{c}{\Gamma (\alpha )}(t-a+1)^{\overline{\alpha -1}}+\frac{1}{\Gamma (\alpha )}\sum _{s=a+1}^{t}(t-s+1)^{\overline{\alpha -1}} g(s). $$

It follows from (21) that

22 $$\begin{aligned} \Gamma (\alpha )(t-a)^{1-\alpha }x(t)&\le c(t-a+1)^{\overline{\alpha -1}}(t-a)^{1-\alpha }\\ &\quad +(t-a)^{1-\alpha }\sum _{s=a+1}^{t}(t-s+1)^{\overline{\alpha -1}} g(t).\end{aligned}$$

By using the Stirling’s formula (Alzabut and Abdeljawad 2014)

$$ \lim _{t\rightarrow \infty }\frac{\Gamma (t)t^{\varepsilon }}{\Gamma (t+\varepsilon )}=1, \quad \varepsilon >0, $$

we obtain

23 $$\begin{aligned} &\lim _{t\rightarrow \infty }(t-a)^{1-\alpha }(t-a+1)^{\overline{\alpha -1}}\\ &\quad =\lim _{t\rightarrow \infty }(t-a)^{1-\alpha } \frac{\Gamma (t-a+1+\alpha -1)}{\Gamma (t-a+1)}\\ &\quad =\lim _{t\rightarrow \infty }(t-a)^{1-\alpha }\frac{\Gamma (t-a+\alpha )}{(t-a)\Gamma (t-a)}\\ &\quad =\lim _{t\rightarrow \infty }\frac{\Gamma (t-a+\alpha )}{(t-a)^{\alpha }\Gamma (t-a)}\\ &\quad =1. \end{aligned}$$

Noting (23) and taking $t\rightarrow \infty$ in (22), we have

24 $$ \liminf _{t\rightarrow \infty }\left\{ (t-a)^{1-\alpha }x(t)\right\} \le -\infty , $$

which contradicts with $x(t)>0$.

### *Case 2*

$x(t)<0, \ t\ge t_0.$ Noting the assumption (A), from the system (1) , we have

$$ \nabla _a^{\alpha }x(t)=-q(t)f(x(t))+g(t)\ge g(t). $$

By Lemma 6, from (24), we obtain

25 $$\nabla _{a+1}^{-\alpha }\nabla _a^{\alpha }x(t)\ge \nabla _{a+1}^{-\alpha }g(t). $$

Using the procedure of Case 1, it follows from (25) that

26 $$\begin{aligned} \Gamma (\alpha )(t-a)^{1-\alpha }x(t)&\ge c(t-a+1)^{\overline{\alpha -1}}(t-a)^{1-\alpha }\\ &\quad +(t-a)^{1-\alpha }\sum _{s=a+1}^{t}(t-s+1)^{\overline{\alpha -1}} g(t).\end{aligned}$$

Noting (23) and taking $t\rightarrow \infty$ in (26), we have

$$ \limsup _{t\rightarrow \infty }\left\{ (t-a)^{1-\alpha }x(t)\right\} \ge +\infty , $$

which contradicts with $x(t)<0$. This completes the proof of Theorem 7.

### **Theorem 8**

Assume that there exists $t_0\in {\mathbb {N}}_{a+1}$ such that

27 $$ \liminf _{t\rightarrow \infty }\left\{ \sum _{s=t_0+1}^{t}g(s)\right\} =-\infty , $$

and

28 $$\limsup _{t\rightarrow \infty }\left\{ \sum _{s=t_0+1}^{t}g(s)\right\} =+\infty . $$

Then every solution *x*(*t*) of the system (1) is oscillatory.

### Proof

Suppose to the contrary that there is a nonoscillatory solution *x*(*t*) of system (1). It is obvious that there exists $t_0\in {\mathbb {N}}_{a+1}$ such that $x(t)>0$ or $x(t)<0, \ t\ge t_0.$

### *Case 1*

$x(t)>0, \ t\ge t_0.$ As in the proof of Theorem 7, we obtain (17). Using Lemma 5, it follows from (17) that

29 $$\nabla E(t)\le \Gamma (1-\alpha )g(t). $$

Summing both sides of (29) from $t_0+1$ to *t*, we have

30 $$ E(t)\le E(t_0)+\Gamma (1-\alpha )\sum _{s=t_0+1}^{t} g(t).$$

Letting $t\rightarrow \infty$ in (30), we obtain

$$\liminf _{t\rightarrow \infty }E(t)=-\infty , $$

which contradicts with $E(t)>0$.

### *Case 2*

$x(t)<0, \ t\ge t_0.$ As in the proof of Theorem 7, we obtain (24). Then, using the above mentioned method, we easily obtain a contradiction. This completes the proof of Theorem 8.

## Remarks

In our Definition 1, the fractional sum in (2) starts at *a*. In Abdeljawad and Atici (2012), Abdeljawad and Atici introduced the following fractional sum.

Let $\nu >0$. The $\nu$-th fractional sum *f* is defined by

31 $$ \widetilde{\nabla }_a^{-\nu }f(t) =\frac{1}{\Gamma (\nu )}\sum _{s=a+1}^{t}(t-s+1)^{\overline{\nu -1}}f(s), \quad t\in {\mathbb {N}}_{a+1}.$$

Obviously, the fractional sum in (31) starts at $a+1$. In Abdeljawad and Atici (2012), the authors established the relation between the operators $\nabla _a^{-\nu }$ and $\widetilde{\nabla }_a^{-\nu }$ and considered the following initial value problem for a nonlinear fractional difference equation

$$ \left\{ \begin{array}{lll} \nabla _{a-1}^{\alpha }x(t)=f(t,x(t)), &{}\quad \text{ for } \ \ t=a+1,a+2,\ldots ,\\ \widetilde{\nabla }_{a-1}^{-(1-\alpha )}x(t)|_{t=a}=x(a)=c, \end{array}\right. \quad \qquad \qquad \qquad (E5) $$

where $0<\alpha <1$ and *a* is any real number. The authors obtained that *x*(*t*) is a solution of (*E*5) if and only if

$$ x(t)=\frac{(t-a+1)^{\overline{\alpha -1}}}{\Gamma (\alpha )}x(a)+\widetilde{\nabla }_a^{-\alpha }f(t,x(t)).$$

Using the idea in Abdeljawad and Atici (2012), we can try to investigate the oscillation of the following nonlinear fractional nabla difference system

$$\left\{ \begin{array}{ll} \nabla _{a-1}^{\alpha }x(t)+q(t)f(x(t))=g(t), &{}\quad t\in {\mathbb {N}}_a,\\ \widetilde{\nabla }_{a-1}^{-(1-\alpha )}x(t)|_{t=a}=c.\end{array}\right. \qquad \qquad \qquad \qquad\qquad (E6) $$

## Examples

In this section, we give some examples to illustrate our main results.

### Example 9

Consider the following fractional nabla difference system

32 $$ \left\{ \begin{array}{ll} \nabla _1^{\frac{1}{2}}x(t)+\frac{\left( t-\frac{\sqrt{\pi }}{2}\right) \Gamma (t)}{\Gamma \left( t+\frac{1}{2}\right) }x(t)=t, &{}\quad t\in {\mathbb {N}}_2,\\ \nabla _1^{-\frac{1}{2}}x(t)\Big |_{t=1}=\frac{\sqrt{\pi }}{2}.\end{array} \right. $$

Here $a=1,\alpha =\frac{1}{2}, q(t)=\frac{(t-\frac{\sqrt{\pi }}{2})\Gamma (t)}{\Gamma (t+\frac{1}{2})}, \ f(x(t))=x(t), \ g(t)=t$. It is easy to see that

33 $$ (t-1)^{\frac{1}{2}}\sum _{s=2}^{t}(t-s+1)^{\overline{-\frac{1}{2}}}g(s) =(t-1)^{\frac{1}{2}}\sum _{s=2}^{t}(t-s+1)^{\overline{-\frac{1}{2}}}s>0, \quad t\in {\mathbb {N}}_2. $$

Therefore, from (33), we obtain that the condition (15) of Theorem 7 does not hold. Indeed, using Lemma 4 and Definition 1, by careful calculation, we find that $x(t)=t^{\overline{\frac{1}{2}}}>0$ is a nonoscillatory solution of system (32).

### Example 10

Consider the following fractional nabla difference system

34 $$ \left\{ \begin{array}{ll}\nabla _1^{\frac{1}{3}}x(t)+\frac{t^2\Gamma (t)}{\Gamma \left( t+\frac{1}{3}\right) }x(t)=t^2+\frac{1}{3}\Gamma \left( \frac{1}{3}\right) , &{}\quad t\in {\mathbb {N}}_2,\\ \nabla _1^{-\frac{2}{3}}x(t)\Big |_{t=1}=\frac{1}{3}\Gamma \left( \frac{1}{3}\right) .\end{array} \right. $$

Here $a=1,\alpha =\frac{1}{3}, \ q(t)=\frac{t^2\Gamma (t)}{\Gamma (t+\frac{1}{3})}, \ f(x(t))=x(t), \ g(t)=t^2+\frac{1}{3}\Gamma \left( \frac{1}{3}\right)$. Obviously, for $t_0\in {\mathbb {N}}_2,$

35 $$\sum _{s=t_0+1}^{t}g(s)=\sum _{s=t_0+1}^{t}\left( s^2+\frac{1}{3}\Gamma \left( \frac{1}{3}\right) \right) >0, $$

which shows that the condition (27) of Theorem 8 does not hold. In fact, we can verify that $x(t)=t^{\overline{\frac{1}{3}}}>0$ is a nonoscillatory solution of system (34).

### Example 11

Consider the following fractional nabla difference system

36 $$ \left\{ \begin{array}{ll}\nabla _1^{\frac{1}{2}}x(t)+\frac{3t}{\Gamma \left( t+\frac{1}{4}\right) }x(t)=(-1)^te^t-(-1)^{t-1}e^{t-1}, &{}\quad t\in {\mathbb {N}}_2,\\ \nabla _1^{-\frac{1}{2}}x(t)\Big |_{t=1}=c_1, &{}\quad (c_1 \text{ is } \text{ a } \text{ constant }).\end{array} \right. $$

Here $a=1,\alpha =\frac{1}{2}, \ q(t)=\frac{3t}{\Gamma (t+\frac{1}{4})} \ f(x(t))=x(t), \ g(t)=(-1)^te^t-(-1)^{t-1}e^{t-1}$. We easily see that

$$ \sum _{s=t_0+1}^tg(s)=(-1)^te^t-(-1)^{t_0}e^{t_0}, \quad t_0\in {\mathbb {N}}_2. $$

Therefore,

37 $$ \liminf _{t\rightarrow \infty }\left\{ \sum _{s=t_0+1}^{t}g(s)\right\} =\liminf _{t\rightarrow \infty }\left\{ (-1)^te^t-(-1)^{t_0}e^{t_0}\right\} =-\infty , $$

and

38 $$ \limsup _{t\rightarrow \infty }\left\{ \sum _{s=t_0+1}^{t}g(s)\right\} =\limsup _{t\rightarrow \infty }\left\{ (-1)^te^t-(-1)^{t_0}e^{t_0}\right\} =+\infty , $$

which show that the conditions in Theorem 8 are satisfied. By Theorem 8, every solution *x*(*t*) of the system (36) is oscillatory.

## Conclusions

This paper provides some oscillation criteria for solutions of a nonlinear fractional nabla difference system by using the basic theories of discrete fractional calculus. The main results are given in Theorems 7 and 8. In the end of this paper, we give three examples. Examples 9 and 10 show that the assumptions of the main results can not be dropped.
